# Heart rate, anxiety and performance of residents during a simulated critical clinical encounter: a pilot study

**DOI:** 10.1186/1472-6920-14-153

**Published:** 2014-07-27

**Authors:** Samuel Clarke, Timothy Horeczko, Dale Cotton, Aaron Bair

**Affiliations:** 1 Department of Emergency Medicine, UC Davis School of Medicine, 4150 V St., PSSB 2100, Sacramento, CA 95817, USA; 2 Department of Emergency Medicine, Harbor-UCLA Medical Center, 1000 W Carson St., Box 21, Torrance, CA 90509, USA; 3 Department of Emergency Medicine, Kaiser Permanente South Sacramento, 6600 Bruceville Road, Sacramento, CA 95823, USA

## Abstract

**Background:**

High-fidelity patient simulation has been praised for its ability to recreate lifelike training conditions. The degree to which high fidelity simulation elicits acute emotional and physiologic stress among participants – and the influence of acute stress on clinical performance in the simulation setting – remain areas of active exploration. We examined the relationship between residents’ self-reported anxiety and a proxy of physiologic stress (heart rate) as well as their clinical performance in a simulation exam using a validated assessment of non-technical skills, the Ottawa Crisis Resource Management Global Rating Scale (Ottawa GRS).

**Methods:**

This was a prospective observational cohort study of emergency medicine residents at a single academic center. Participants managed a simulated clinical encounter. Anxiety was assessed using a pre- and post-simulation survey, and continuous cardiac monitoring was performed on each participant during the scenario. Performance in the simulation scenario was graded by faculty raters using a critical actions checklist and the Ottawa GRS instrument.

**Results:**

Data collection occurred during the 2011 academic year. Of 40 eligible residents, 34 were included in the analysis. The median baseline heart rate for participants was 70 beats per minute (IQR: 62 – 78). During the simulation, the median maximum heart rate was 140 beats per minute (IQR: 137 – 151). The median minimum heart rate during simulation was 81 beats per minute (IQR: 72 – 92), and mean heart rate was 117 beats per minute (95% CI: 111 – 123). Pre- and post-simulation anxiety scores were equal (mean 3.3, IQR: 3 to 4). The minimum and maximum *Overall* Ottawa GRS scores were 2.33 and 6.67, respectively. The median *Overall* score was 5.63 (IQR: 5.0 to 6.0). Of the candidate predictors of *Overall* performance in a multivariate logistic regression model, only PGY status showed statistical significance (*P* = 0.02).

**Conclusions:**

Simulation is associated with physiologic stress, and heart rate elevation alone correlates poorly with both perceived stress and performance. Non-technical performance in the simulation setting may be more closely tied to one’s level of clinical experience than to perceived or actual stress.

## Background

Simulation training has become an integral component of medical education in both undergraduate and graduate medical education settings [1,2]. Most rapidly adopted in acute care specialties, high-fidelity training manikins are used in approximately 90% of emergency medicine residencies [3]. Simulation can offer a safe training environment to practice high-risk/low-frequency medical scenarios and engage learners on multiple levels: cognitive, procedural, and affective [4].

Simulation can provide realistic training scenarios that may evoke psychological stress similar to that of actual medical emergencies. The extent to which acute stress during a simulation activity may augment or diminish performance and learning however, remains unclear [5]. Experiential learning requires some degree of engagement and stress; DeMaria and colleagues [6] found that a simulation scenario that induced anxiety led to better performance and retention of ACLS skills among medical students than one that did not. LeBlanc et al. [7] studied first-year surgical residents and found that moderate stress levels facilitated performance of technical procedures. If a minimum level of stress is beneficial, there may exist a personal threshold beyond which situational stress is detrimental. Hunziker et al. [8] found that medical students performing CPR in a simulation exercise demonstrated high levels of emotional overload and significantly poorer clinical performance. Two additional studies by LeBlanc et al. [9,10] found that paramedics exposed to high levels of acute stress during simulation scenarios were more likely to commit errors in communication and medication dosing.

High levels of stress likely impair cognitive performance through inhibition of declarative and working memory and the ability to perform tasks involving divided attention [7]. Cognitive appraisal theory posits that an individual’s response to demands that threaten an important goal (e.g. successfully navigating a clinical scenario) involve an interplay between the individual’s perception of the demands and the resources available to meet them [7]. Demands which are sufficiently met by available resources (both internal and external) are perceived positively as challenges; those which outstrip available resources are perceived as threats. Studies of physiologic response have shown correlation between individuals’ cognitive appraisal of a situation as either threatening or challenging (i.e. negative or positive) with salivary cortisol levels as well as heart rate [5,11]. The "real world" application of this physiologic understanding in the learning environment remains elusive.

A number of previous studies have assessed the relationship between stress (both physiologic and emotional) and performance of basic technical skills (e.g. CPR performance and medication dosing), but few have addressed the impact of physiologic stress on non-technical skills relevant to clinical performance [12]. Crisis resource management (CRM) refers to the constellation of non-technical skills (e.g. leadership, situational awareness, communication) that comprise effective team performance [13,14]. A recent systematic review of the relationship between non-technical and technical skills in the operative environment found that failures in non-technical skills are associated with higher rates of technical errors [15].

We sought to determine the global degree of physiologic stress experienced by various levels of emergency medicine trainees via self-assessments and continuous measurement of heart rate and rhythm throughout a given simulation scenario, and to assess clinical performance using a validated assessment instrument of crisis resource management (CRM) ability, the Ottawa Global Rating Scale (GRS). The purpose of our study was to determine whether high degrees of self-reported stress prior to a simulation exercise correlate with heart rate elevation during the exercise. Our secondary outcome of interest was to determine whether heart rate elevation as a proxy for stress correlates with CRM ability in the simulation environment. We hypothesized that residents who reported higher degrees of stress prior to the simulation scenario would be more likely to manifest physiologic evidence of that stress with changes in heart rate during medical simulation. Further, we hypothesized that higher levels of perceived stress (as a proxy for psychological stress) and higher heart rates (as a proxy for physiologic stress) would vary with PGY status. The purpose of this study was to identify candidate factors that may be predictive of overall CRM performance as measured by the Ottawa GRS, and to determine the relationship between physiologic stress and clinical performance using the percentage of critical actions (termed the "Flow Score") performed in each simulation scenario.

## Methods

This was a prospective observational cohort study conducted in the Department of Emergency Medicine at UC Davis Medical Center between October and December of 2011. This research study was deemed exempt by the UC Davis IRB. Our emergency medicine residency is an Accreditation Council for Graduate Medical Education (ACGME)-accredited, post-graduate year (PGY)-1 through PGY-3 program. Our residents spend 70% of their training time at UC Davis Medical Center, a Level-I Trauma Center with an Emergency Department census of approximately 70,000 visits per year, and the remaining 30% at a community based Level-2 Trauma Center with an annual patient census of 90,000 visits per year.

The residency program has used an annual simulation exam since 2006. Participation in the simulation exam is considered mandatory; residents who perform below expectations on the exam are scheduled for a repeat examination and special session with a faculty member to review performance. Each resident independently manages the resuscitation of a simulated critically ill patient. The level of difficulty of the simulation cases is stratified by post-graduate year: PGY-1 residents manage an ACLS-themed case involving a patient in cardiac arrest; PGY-2 residents manage a patient with sepsis and respiratory failure; and PGY-3 residents manage a case involving a critically ill polytrauma patient (Additional files 1, 2 and 3). A group of 3-5 faculty raters assessed each resident’s performance using a critical actions checklist (i.e. the "Flow Score", or percentage of critical actions performed correctly in a given simulation, see Additional files 1, 2 and 3) and the Ottawa Crisis Resource Management Global Rating Scale (Ottawa GRS), a validated instrument of crisis leadership and communication ability (Additional file 4). The raters each filled out their evaluations independently from each other. All raters were members of our Department of Emergency Medicine faculty and were therefore not blinded to the study participants’ PGY-status. The simulation cases were developed and vetted by members of our faculty with expertise in simulation, and had been used for multiple years prior to our study.

The 2011 simulation exam served as the setting for our study. All participants enrolled in the study were current residents within our program.

We assessed resident performance in the simulation scenario in two ways. Each resident performed a PGY-level specific simulation case and was graded on performance of critical actions using the Flow Score. The Flow Score, an adjunct measurement used by the department’s simulation experts, is analogous to the traditional percentage "grade" in a test. That is, a Flow Score of 0.9 indicates that the resident responded correctly to 90% of the possible items in a given scenario.

Leadership and communication behaviors were graded using the Ottawa GRS instrument. The Ottawa GRS consists of 5 domains relating to crisis resource management (CRM) ability (*Leadership, Problem Solving, Situational Awareness, Resource Utilization,* and *Communication*), as well as an *Overall* score. Each domain uses a 7-point Likert-style scale with descriptive anchors to aid in scoring. For our analysis we assessed performance with the *Overall* domain, as this is a summative score most likely to be representative of a given rater’s global impression of resident CRM ability. In the interest of robust reporting, the highest and lowest faculty ratings of the Flow Score and *Overall* Ottawa GRS score were truncated and the remaining scores were averaged (modified mean).

We assessed residents’ level of anxiety in the following manner: immediately prior to the simulation session, participants completed a questionnaire asking them to rate their anxiety level on a 5 point scale with 1 being "no stress" and 5 being "very stressed" (Additional file 5). The questionnaire was developed by the study investigators specifically for this study. Participants were also queried on their average caffeine consumption and consumption in the preceding 12 hours, as well as the use of medications (e.g. beta blockers or over-the-counter cough-and-cold preparations) which might affect heart rate. All participants rated their level of anxiety again immediately after the simulation session.

We chose heart rate as a measure of physiologic stress. We attached a standard 5-lead Holter monitor to each study participant immediately prior to the simulation exercise, and performed continuous recording throughout the simulation. Minimum, maximum, and mean heart rates, as well as alerts for premature ventricular contractions (PVCs) or other dysrhythmias were electronically abstracted by a Holter technician, and reviewed by the study investigators.

We calculated descriptive statistics for participants’ heart rate and anxiety levels as well as their corresponding Flow Scores and Ottawa GRS scores. We then constructed an investigator-built multivariate logistic regression model to identify independent variables that may predict the dependent variable, the *Overall* Ottawa GRS Score. Given our sample size, we limited this regression analysis to accommodate the guidelines of at least 10 observations per candidate variable [16]. Dependent variables for this model included: pre-test anxiety level, mean heart rate, peak heart rate, and PGY status (see Table 1 for full model specifications). All statistical analyses were performed using SAS software version 9.3 (SAS Institute Inc., Cary, North Carolina).

**Table 1:** Multivariate regression model for resident performance using physiologic parameters

	**Estimate**	**SE**	**t value**	**P**
Intercept	6.38204	1.59887	1.59887	1.59887
Pre-test anxiety level	-0.10412	0.19231	-0.54	0.5925
Mean heart rate	-0.01918	0.01926	-1	0.3279
Peak heart rate	0.00924	0.01873	0.49	0.6256
PGY status	0.52198	0.20129	2.59	0.015

Model: Ottawa Overall Score = *β*_*0*_ + *β*_*1*_(pre-test anxiety level) + *β*_*2*_(mean heart rate) + *β*_*3*_(peak heart rate) + *β*_*4*_(PGY status) + *e.*

Adjusted R-square for model = 0.16; F = 2.48; P = 0.07.

## Results

Of the 40 eligible participants, 34 were included in our analysis. One resident declined participation in the study, two were excluded for recent medication use, and three were excluded due to incomplete or uninterpretable Holter monitor data (i.e. due to equipment failure or placement error). Of the 34 study participants, 13 were female (Table 2). There were 13 PGY-1 level participants, 13 PGY-2 level participants, and 8 PGY-3 level participants. The median age of participants was 29 years (IQR 28 – 31). Sixty-eight percent of participants (n = 23) reported consumption of at least one caffeinated beverage in the 12 hours preceding the simulation session; roughly half reported consuming caffeine on a regular basis.

**Table 2:** Baseline characteristics of resident participants; N = 34

Age — years (IQR)	29 (28 to 31)
Gender — n (%)	
Female	13 (38)
Male	21 (62)
PGY status — n (%)	
1st year resident	13 (38)
2nd year resident	13 (38)
3rd year resident	8 (24)
Usual daily caffeine intake* — n (%)	
Less than one cup	11 (32)
One to three cups	19 (56)
Three or more cups	4 (12)
Caffeine intake* on simulation day — n (%)	
Less than one cup	15 (44)
One to three cups	16 (47)
Three or more cups	3 (8)
Baseline heart rate^†^ — median beats per min (IQR)	70 (62 to 78)

*Expressed in cups of regular coffee or its equivalent (i.e. 100 mg caffeine).

^†^Measured during academic conference, not on simulation day.

The median baseline heart rate for participants, obtained at rest during a non-simulation based academic event, was 70 beats per minute (IQR: 62 – 78) (Table 3). During the simulation, the median maximum heart rate was 140 beats per minute (IQR: 137 – 151). The median minimum heart rate during simulation was 81 beats per minute (IQR: 72 – 92), and mean heart rate was 117 beats per minute (95% CI: 111 – 123). Ventricular ectopy was common during the simulation exercise: 8 residents experienced 1 to 3 premature ventricular contractions (PVCs), and one resident displayed 28 beats of bigeminy and was subsequently referred for further evaluation.

**Table 3:** Physiologic parameters and resident performance; N = 34

Self-reported level of anxiety* — rating (IQR)	
Pre-test	3.3 (3 to 4)
Post-test	3.3 (3 to 4)
Minimum heart rate — median (IQR)	81 (72 to 92)
Heart rate — median (IQR)	115 (107 to 127)
Maximum heart rate — median (IQR)	140 (137 to 151)
Ectopy on Holter monitor — n (%)	
0 PVC	25 (73.5)
1 PVC	6 (17.7)
2 PVCs	1 (2.9)
3 PVCs	1 (2.9)
28 PVCs	1 (2.9)
Fraction of items correct — median Flow Score^†^ (IQR)	0.92 (0.86 to 0.95)

*Level of anxiety on a scale of 0 (no anxiety) to 5 (most anxious).

^†^Flow Score is the fraction of items correct/total items (0 to 1).

Pre- and post-simulation anxiety scores were equal (mean 3.3, IQR: 3 to 4). The minimum and maximum Flow Scores were 0.59 and 0.99, respectively. The median Flow Score was 0.92 (IQR: 0.86 to 0.95).

The minimum and maximum *Overall* Ottawa GRS scores were 2.33 and 6.67, respectively. The median *Overall* score was 5.63 (IQR: 5.0 to 6.0).

**Figure 1:**
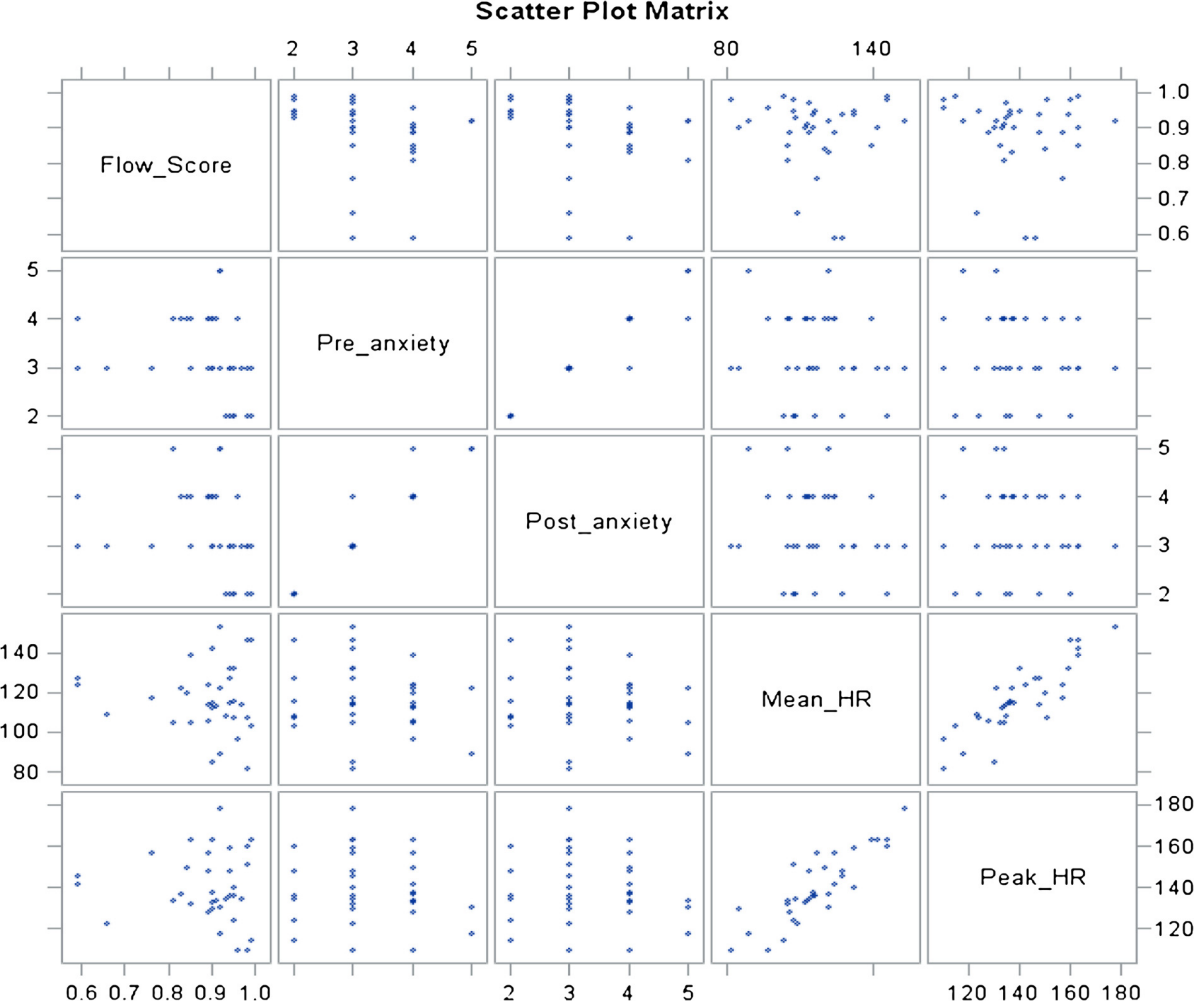
Correlation of candidate predictor variables with simulation flow score.

**Figure 2:**
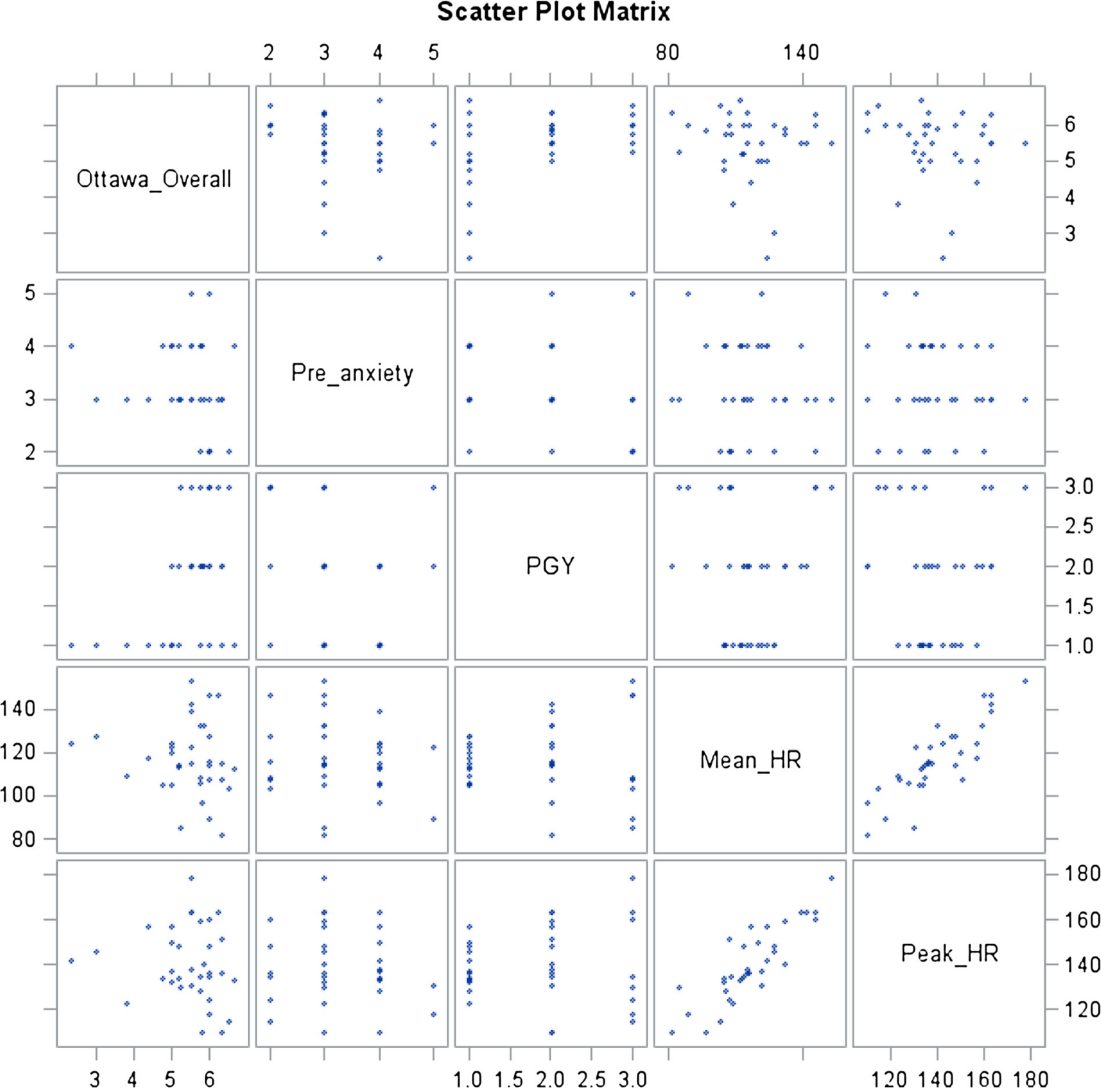
Correlation of candidate predictor variables with overall Ottawa GRS.

Individual correlations of candidate predictors and the dependent variables of Flow Score and *Overall* Ottawa GRS Score are shown in scatter matrices in Figures 1 and 2. The visual representation of correlations of pre- and post-anxiety with Flow Score was similar; in addition, pre-anxiety correlated well with post-anxiety. Peak heart rate and mean heart rate showed similar relationships. However, pre-anxiety and mean and peak heart rates did not appear to have a discernible relationship. In terms of peak and mean heart rates, the lower values tended to cluster around higher Flow Scores. *Overall* Ottawa GRS Score showed a trend of homogenization with increasing PGY status: PGY status showed both an increase in *Overall* score as well as a more uniform performance by group (Figure 2). Neither Overall Ottawa GRS score nor Flow Score appeared to correlate with mean or peak heart rates (Figures 1 and 2).

Of the candidate predictors of *Overall* performance in the multivariate logistic regression model, only PGY status showed statistical significance (*P* = 0.02) in this limited model. Regarding performance of this particular model as a whole, the adjusted pseudo R-squared was not statistically significant (P = 0.07) and at 16% not explanatory (100% indicating that the model explains all of the variability of the response data around its mean) (Table 1).

## Discussion

In this exploratory study, we examined the relationship between self-reported anxiety and a measure of physiologic stress (heart rate) in relation to clinical performance among emergency medicine residents performing a simulation test. We furthermore sought to determine whether anxiety level and clinical performance would vary according to PGY-status. While we found no relationship between pre-test anxiety and mean or peak heart rate (our primary hypothesis), we found that the simulation exercise was associated with significant heart rate elevation, and in one case sustained ventricular ectopy.

A previous study conducted by Harvey et al. examined subjective and physiologic responses among residents performing simulation scenarios under varying stress levels [12]. While participation in both high- and low-stress simulation scenarios was associated with increased heart rate compared to baseline, they found no significant difference in heart rate elevation between the high and low-stress conditions, while cortisol levels and subjective stress measurement were significantly higher in the high-stress scenario. A study of CPR performance by Hunziker et al. similarly found that perceived stress was inversely correlated with heart rate variability (a finding observed elsewhere in studies of physiologic stress response), but showed no significant relationship with heart rate elevation [17,18]. And a study by Girzadas et al. found no relationship between heart rate elevation and perceived stress among residents performing airway simulations [19]. The relationship between emotional and physiologic anxiety states is complex, and involves the interplay of the neurohormonal axis, circadian rhythms, and individual variability in chronotropic response to stress [20]. As such, heart rate elevation alone may be an incomplete measure of perceived stress.

With regard to the relationship between perceived stress, PGY-status, and performance, only PGY-status correlated with the Ottawa GRS *Overall* score in our multivariate logistic regression model. This finding is consistent with our institutional experience that residents’ cumulative exposure to critically ill patients in simulated and clinical environments correlates with improvement in self-assessed and faculty-assessed performance.

Quantifying perceived stress and its corresponding physiologic response is a subject of ongoing investigation. We observed no difference in self-reported anxiety among participants prior to and after the simulation experience. While at first glance surprising, we suspect this may reflect our using a relatively broad survey item to assess participants’ feelings (a simple Likert-style question addressing global anxiety about the simulation experience). A more detailed survey with serial longitudinal assessments may have captured more nuanced levels of self-reflection among participants. A previous study of emergency medicine residents found that senior residents’ performance was less influenced by stressful testing conditions than that of junior residents, although this same study found that junior residents’ performance actually improved under moderately stressful conditions [21]. While there is ample evidence to suggest that high-fidelity simulation can evoke emotional and physiologic stress, it may ultimately be the learner’s perception of that stress that predicts performance. The "mixed picture" of stress and performance in previous studies is likely attributable to a failure to recognize individual learners’ feelings of ability to meet the demands imposed by a given scenario. Acute stress, when overwhelming, may inhibit cognitive performance. At lower levels, however, it may in fact facilitate motivation, task awareness, and concentration [21]. It is likely that the relationship between stress and performance involves the interplay of intrinsic factors, such as one’s level of experience and perception of task-related demands versus resources, and extrinsic factors such as the complexity of the task and the nature and intensity of related stressors.

Our study has a number of important limitations. It was performed at a single institution, and as such may strongly reflect the training environment and instructional methods of our program. We attempted to mitigate this limitation with standardized scenarios and the widely used Ottawa GRS. Given our small sample size, excluded participants (15%), and number of candidate predictors, we present a limited predictive model; a larger study may produce a more comprehensive model that predicts clinical performance with finer precision. We also used a subjective measure of self-reported anxiety via a non-validated survey instrument; our findings may therefore not reflect subtler motivational and perceptual aspects of anxiety and performance. Due to clinical time obligations of the residents, in depth psychometric testing pre- and post-simulation was not a feasible option. Finally, our study involved faculty members from our own residency program to assess resident performance: our raters were not blinded to participants’ PGY-status and may have been influenced by prior shared experience in the clinical environment.

## Conclusions

This exploratory study supports previous findings that simulation is associated with physiologic stress, and that heart rate elevation alone correlates poorly with both perceived stress and performance. Our study suggests that non-technical performance in the simulation setting may be more closely tied to one’s level of clinical experience than to perceived or actual stress. Further research into the motivational and perceptual aspects of acute stress is needed to elucidate the complex relationship between perceived demand, available resources, level of experience, physiologic stress, and clinical performance as measured in the simulation environment.

## Abbreviations

Ottawa GRS: Ottawa Crisis Resource Management Global Rating Scale; CRM: Crisis resource management; ACGME: Accreditation Council for Graduate Medical Education; PGY: Post-graduate year; PVC: Premature ventricular contraction.

## Competing interests

The authors declare that they have no competing interests.

## Authors’ contributions

SC participated in the interpretation of results and was the primary author of the manuscript. TH performed the statistical analysis and assisted in drafting and editing the manuscript. DC conceived of the study and coordinated the data collection. AB assisted in the study design, data collection and assisted in editing the manuscript. All authors read and approved the final manuscript.

## Pre-publication history

The pre-publication history for this paper can be accessed here:

http://www.biomedcentral.com/1472-6920/14/153/prepub

## Supplementary Material

Additional file 1 Emergency Residents Assessment Scenario – R1 "Ventricular Fibrillation". Click here for file

Additional file 2 Emergency Residents Assessment Scenario – R2 "Septic Shock". Click here for file

Additional file 3 Emergency Residents Assessment Scenario – R3 "Poly Trauma". Click here for file

Additional file 4 Ottawa Crisis Resource Management (CRM) Global Rating Scale ("Ottawa GRS"). Click here for file

Additional file 5 Anxiety and Caffeine Consumption Questionnaire. Click here for file
