# Supplementary material for: Heart rate, anxiety and performance of residents during a simulated critical clinical encounter: a pilot study
Source: BMC Med Educ. 2014 Jul 27;14:153. doi: 10.1186/1472-6920-14-153 (PMC4131479; doi:10.1186/1472-6920-14-153)
Supplement: Additional file 1 — Emergency Residents Assessment Scenario – R1 "Ventricular Fibrillation". [file 1472-6920-14-153-S1.docx]

**Additional file 1: Emergency Residents Assessment Scenario – R1 “Ventricular Fibrillation”**

**Case Details:**

A 65 year-old male, brought in by ambulance with the chief complaint of chest pain for 2 hours. The pain has been intermittent for the past 3 weeks. The patient stated **“this pain is similar to the pain I had few years ago when I was diagnosed with a heart attack”.** The pain started upon waking, the pain is score 10/10, substernal, associated with nausea and vomiting. It is radiating to the left arm and jaw. Now, the pain is less in intensity 5/10.

**Past Medical History:** Myocardial Infarction, Diabetes Mellitus, Hypertension, Hyperlipidemia

**Medications:** Metformin, Lipitor, Maxzide

**Past Social History:** Left Total Knee (years ago)

**Family Social History:** Coronary Artery Disease; smoker for the past 40 years, married, retired

**Allergies:** None

| **Case Progression** | **Goals** | **Critical Actions** |
| --- | --- | --- |
| On arrival to the Emergency Department | - Identify that the Chest Pain patient is a priority patient - Rapidly assess the potentially critical patient (“Medical Red”) - Recognize “typical” cardiac ischemia symptoms - Obtain History - Elicit drug allergies - Get EMS report - Identify as priority patient (get nursing and tech support) | - Perform focused physical exam - Obtain 12-lead ECG - Place on oxygen - Start IVs - Place on monitor (including SpO_2_) |
| **Physical Exam:**  **Blood Pressure:** 160/90 mmHg, **Heart Rate:** 110 beats/minute, **Respiratory Rate:** 20 breath/ minute, **SpO_2:_** 100% on 4L NC  **General Appearance:** the patient is awake/Alert, Anxious and Diaphoretic  **Lungs:** clear  **Heart:** tachycardia, regular heart rate and no murmur  **Perfusion**: good  **Abdomen:** soft and no organomegaly  **EMS Report**: Chest Pain protocol started.  Given sublingual Nitroglycerin NTG 0.4 mg 3 times  Chest Pain score 10/10🡪6/10  Blood Pressure 190/110🡪150/90  Patient refuses Aspirin (GI upset)  **1^st^ 12-lead: Anterior ST segment Elevation Myocardial Infarction (STEMI)** | - Identify cardiac Ischemia/Infarction - Differentiate medication intolerance from true allergy - Recognize the need for rapid intervention in Acute Coronary Syndrome (ACS)/ STEMI - Cardiology Consult | - Portable Chest X-Ray - Administer appropriate medications: Aspirin, Nitroglycerine, morphine, heparin - Reperfusion therapy Cardiac Catheterization vs. Thrombolytic - Reassess after interventions (pain score and vital signs) |
| Patient becomes unresponsive  Eyes roll back  No movement  **Monitor shows:** Ventricular Fibrillation (VF) | - Identify pulseless arrest - Differentiate VF from stable rhythms - Assume leadership role directing “code” - Recognize VF requires rapid intervention (defibrillation) - Use the correct ACLS algorithm for pulseless rhythms - Adequate CPR | - Start CPR immediately - Appropriate defibrillation - Provide a BLS airway - Resume CPR immediately after shock (for 2 min. or 5 cycles) - Appropriate medications administration: Epinephrine or Vasopressin during compressions |
| **At two minutes:** CPR stops and patient remains in VF  No pulse  Patient is ashen and mottled  Vomitus in the airway | - Recognize pulseless rhythm - Recognize shockable rhythm - Recognize the need for advanced airway | - Intubate & confirm tube placement - Appropriate ongoing CPR and shocks - Appropriate antiarrhythmic (i.e. Lidocaine or Amiodarone) |
| **At two minutes:** CPR stops monitor shows sinus rhythm: 120 beats/ minutes, Blood Pressure: 100/50mmHg, SpO_2_ 94% with bag valve mask.  Patient is agitated | - Recognize Return of Spontaneous Circulation (ROSC) - Recognize hypoxemia as dangerous in coronary ischemia | - Reassess condition - Post intubation management - Portable chest xray |

**End Scenario**
