# Supplementary material for: Heart rate, anxiety and performance of residents during a simulated critical clinical encounter: a pilot study
Source: BMC Med Educ. 2014 Jul 27;14:153. doi: 10.1186/1472-6920-14-153 (PMC4131479; doi:10.1186/1472-6920-14-153)
Supplement: Additional file 2 — Emergency Residents Assessment Scenario – R2 "Septic Shock". [file 1472-6920-14-153-S2.docx]

**Additional file 2: Emergency Residents Assessment Scenario – R2 “Septic Shock”**

**Case Details:**

A 48 year-old male presenting with 3 days history of cough and fevers. The cough is productive of greenish sputum and associated with shortness of breath and pleuritic chest pain. He has mild orthostatic symptoms.

Review of systems is negative except for the previously mentioned symptoms.

**Past Medical History:** Previously healthy, no surgeries. Not on any medications

**Past Social History:** Smoker for the past 30 years, alcohol – “a fair amount”, no illicit drug use. The patient travels extensively in US for work (sales).

**Family Social History:** Married, monogamous. The patient has three school aged children – all have Upper Respiratory Tract Infections (URI).

**Allergies:** None

| **Case Progression** | **Goals** | **Critical Actions** |
| --- | --- | --- |
| On Arrival to the Emergency Department | - Meets Systemic Inflammatory Response Syndrome (SIRS) criteria - Obtain history - Treat patient as priority | - Attach Monitors - Order Chest X-Ray (CXR) |
| **Physical Exam**:  **Heart Rate:** 100 beats/ minute, **Blood Pressure:** 110/70 mmHg, **Respiratory Rate:** 28 breaths/ minutes, **Temperature:** 38.2 ^0^C, **SpO2:** 92% on Room Air (RA), 100% on non-rebreather  **General Appearance:** the patient is awake & alert, using accessory muscle, productive active cough.  **Lungs:** Crackles at right lower base  ***CXR shows Right Lower Lobe Pneumonia*** | - Recognize Sepsis = SIRS plus documented infection - Identify pneumonia - Order antibiotics - Oxygen - Order Labs – CBC/ Chemistry/ lactate/ Arterial Blood Gas (ABG) - Fluid bolus | - Identify pneumonia - Order Appropriate antibiotics - Early Goal Directed Therapy - Oxygen |
| **Twenty minutes later:**  Patient confused  **Heart Rate:** 120 beats/ minutes, **Blood Pressure:** 90/38 mmHg, **Respiratory Rate:** 30 breaths/ minutes,  **SaO_2_** 89% on RA, 97% on NRB  **Labs: Na** 140, **K** 4.5, **Cl** 103, **HCO3** 17, **BUN**19, **creatinine** 1.7,  **Glucose** 121, **Lactate** 5  **WBC** 20,000  **Hct** 37 | - Recognize SEVERE sepsis = sepsis plus at least one sign of organ hypoperfusion (altered mental status and high lactate) - Track urine output - Reassess vital signs | - Identify **metabolic acidosis** - Respiratory support either by intubation or BiPAP - Initiate resuscitation with crystalloid - Notify MICU |
| **Forty minutes later:**  Patient very confused, not compliant  **After 2 Liter (L) bolus:**  Heart Rate: 126 beats/ minutes, Blood Pressure: 90/32 mmHg, Respiratory Rate: 40 breaths/ minutes  **SpO2:** 92% on NRB, 81% on RA  Further 2L (4L total)  **Heart Rate:** 130 beats/ minutes and **Blood Pressure:** 85/25 mmHg | - Need to recognize indication for intubation - Crystalloid fluid bolus 40-60ml/kg (i.e. 3-5L) - Foley catheter - Ventilate at 6-7ml/kg   ~ 400ml tidal volume, PEEP 5   - Consider Inotropic support - **Dopamine** 5 micrograms/kg/min - **Epinephrine /Norepinephrine** 0.25 micrograms/kg/min - Transfusion to keep Hct>30% - Failure to respond to fluid bolus = **septic shock** | - Intubate using appropriate technique - More fluid to reach 50 ml/kg bolus - Place Central Venous Catheter for vasopressors and Scv0_2_   Monitoring   - Inotropic support |
| Inotropic support started  Lactate 6.7  MICU busy- “will be down soon”  Hemodynamic monitoring | - Goal CVP 8-12 mm Hg - Goal MAP 65-90 - Goal ScvO2 >70% | - Post intubation management |

**End Scenario**
