# Supplementary material for: Heart rate, anxiety and performance of residents during a simulated critical clinical encounter: a pilot study
Source: BMC Med Educ. 2014 Jul 27;14:153. doi: 10.1186/1472-6920-14-153 (PMC4131479; doi:10.1186/1472-6920-14-153)
Supplement: Additional file 3 — Emergency Residents Assessment Scenario – R3 "Poly Trauma". [file 1472-6920-14-153-S3.docx]

**Additional file 3: Emergency Residents Assessment Scenario – R3 “Poly Trauma”**

**Case Details:**

A 40 year-old male, brought in by ambulance to Community Hospital. Patient was unrestrained driver involved in a high speed Motor Vehicle Crash (MVC), arrives hypotensive with altered mental status.

He is complaining of headache, chest and abdominal pain. Patient reports loss of consciousness. Systolic Blood Pressure is 80 at scene.

**Past Medical History/Past Social History:** unable to provide

| Case Progression | Goals | Critical Actions |
| --- | --- | --- |
| On arrival to the Emergency Department | - Rapidly assess and address multiple potential causes of hypotension in poly trauma patient | - Obtain AMPLE History - Perform focused physical exam ABCDE and address issues - Start IVs and fluid bolus - Place on monitor |
| **Physical Exam:**  **Blood Pressure:** 110/70 mmHg, **Heart Rate:** 110 beats/minute, **Respiratory Rate:** 30 breaths/ minute, **SpO2:** 100% on 4L Nasal Cannula  **General Appearance:**  Somnolent, intermittently, follows commands  **Lungs:** clear, abrasion across left chest  **Heart**: tachycardia, regular, no murmur  Strong pulses  **Abdomen:** soft with mild diffuse TTP and mid abrasion | - Recognize need for rapid intervention - Identify: - Closed head injury (with decreased level of consciousness) - Small left sided pneumothorax - Hemoperitoneum with splenic rupture (per CT scan) | - Reassess after initial fluid bolus - Appropriate imaging: Chest X-Ray, Pelvis, FAST exam (positive free fluid) |
| Patient becomes less responsive and more hypotensive (90/45 mmHg) | - Identify change | - Check responsiveness - Intubate to protect airway in anticipation of transport (CT scan /OR) - Appropriate use of Rapid Sequence Intubation and dosing of agents |
| No palpable blood pressure after intubation | - Recognize potential causes of post-intubation hypotension - Exclude esophageal intubation - Diagnose tension pneumothorax - Consider medication effect - Consider ongoing hemorrhage | - Appropriate management of post intubation hypotension - Appropriate management of pneumothorax in concert with intubation - Appropriate use of blood products |
| **Repeat vitals:**  **Blood Pressure:** 90/45 mmHg, **Heart Rate:** 110 beats/ minute  **Patient will require transfer to Trauma Center after initial stabilization of intra-abdominal injuries** | - Prioritize disposition | - Contact Surgeon at receiving center - Contact local surgeon for laparotomy to stabilize for transport |

**End Scenario**
