# Supplementary material for: Heart rate, anxiety and performance of residents during a simulated critical clinical encounter: a pilot study
Source: BMC Med Educ. 2014 Jul 27;14:153. doi: 10.1186/1472-6920-14-153 (PMC4131479; doi:10.1186/1472-6920-14-153)
Supplement: Additional file 4 — Ottawa Crisis Resource Management (CRM) Global Rating Scale ("Ottawa GRS"). [file 1472-6920-14-153-S4.pdf]

APPENDIX D: OTTAWA CRISIS RESOURCE MANAGEMENT (CRM) GLOBAL RATING SCALE ("Ottawa GRS")

EVALUATION CRITERIA:

This evaluation scale is directed towards assessing competence in crisis management (CM) skills and care of critically ill patients. The standard of competence has been set at the senior resident level, i.e. the third-year resident who has had prior ICU experience, and through experience as a senior housestaff physician, has previous experience in managing crises. As there exists a requisite base of medical knowledge required to effectively manage crises, this will also be evaluated. However, the focus of evaluation will be on crisis management skills. The skills listed below comprise essential aspects of crisis management. In the simulator case scenario sessions, performance in each of these areas will be assessed, in addition to the amount of prompting or guidance required during the case scenario sessions.

The following criteria will be evaluated:

LEADERSHIP SKILLS
Stays calm and in control during crisis
Prompt and firm decision-making
Maintains global perspective ("Big picture")

SITUATIONAL AWARENESS
Avoids fixation error
Reassesses and re-evaluates situation constantly
Anticipates likely events

COMMUNICATION SKILLS
Communicates clearly and concisely
Uses directed verbal/non-verbal communication
Listens to team input

PROBLEM SOLVING
Organized and efficient problem solving approach (ABC's)
Quick in implementation (Concurrent management)
Considers alternatives during crisis

RESOURCE UTILIZATION
Calls for help appropriately
Utilizes resources at hand appropriately
Prioritizes tasks appropriately

OVERALL

Resident #: ____________________ **Date:** ____________________

Staff: ____________________ **Time:** ____________________

OVERALL PERFORMANCE

1 2	3 4	5 6	7
Novice; all CM skills require significant improvement	Advanced novice; many CM skills require moderate improvement	Competent; most CM skills require minor improvement	Clearly superior; few, if any CM skills that only require minor improvement

I. LEADERSHIP SKILLS

1 2	3 4	5 6	7
Loses calm and control for most of crisis; unable to make firm decisions; cannot maintain global perspective	Loses calm/control frequently during crisis; delays in making firm decisions (or with cueing); rarely maintains global perspective	Stays calm and in control for most of crisis; makes firm decisions with little delay; usually maintains global perspective	Remains cal m and in control for entire crisis; makes prompt and firm decisions without delay; always maintains global perspective

II. PROBLEM SOLVING SKILLS

1 2	3 4	5 6	7
Cannot implement ABC's assessment without direct cues; uses sequential management despite cues; fails to consider any alternative in crisis	Incomplete or slow ABC assessment; mostly uses sequential management approach unless cued; gives little consideration to alternatives	Satisfactory ABC assessment; without cues; mostly uses concurrent management approach with only minimal cueing; considers some alternatives in crisis	Thorough yet quick ABC without cues; always uses concurrent management approach; considers most likely alternatives in crisis

III. SITUATIONAL AWARENESS SKILLS

1 2	3 4	5 6	7
Becomes fixated easily despite repeated cues; fails to re-assess and re-evaluate situation despite repeated cues; fails to anticipate likely events	Avoids fixation error only with cueing; rarely reassesses and re-evaluates situation without cues; rarely anticipates likely events	Usually avoids fixation error with minimal cueing; reassesses re-evaluates situation frequently with minimal cues; usually anticipates likely events	Avoids any fixation error without cues; constantly reassesses and re-evaluates situation without cues; constantly anticipates likely events

IV. RESOURCE UTILIZATION SKILLS

1 2	3 4	5 6	7
Unable to use resources & staff effectively; does not prioritize tasks or ask for help when required despite cues	Able to use resources with minimal effectiveness; only prioritizes tasks or asks for help when required with cues	Able to use resources with moderate effectiveness; able to prioritize tasks and/or ask for help with minimal cues	Clearly able to utilize resources to maximal effectiveness; sets clear task priority and asks for help early with no cues

V. COMMUNICATION SKILLS

1 2	3 4	5 6	7
Does not communicate with staff; does not acknowledge staff communication, never uses directed verbal/non-verbal communication	Communicates occasionally with staff, but unclear and vague; occasionally listens to but rarely interacts with staff; rarely uses directed verbal/ non-verbal communication	Communicates with staff clearly and concisely most of time; listens to staff feedback; usually uses directed verbal/ non-verbal communication	Communicates clearly and concisely at all times, encourages input and listens to staff feedback; consistently uses directed verbal/ non-verbal communication

Reproduced with permission from Lippincott Williams & Wilkins
