# Supplementary material for: Heart rate, anxiety and performance of residents during a simulated critical clinical encounter: a pilot study
Source: BMC Med Educ. 2014 Jul 27;14:153. doi: 10.1186/1472-6920-14-153 (PMC4131479; doi:10.1186/1472-6920-14-153)
Supplement: Additional file 5 — Anxiety and Caffeine Consumption Questionnaire. [file 1472-6920-14-153-S5.doc]

**Additional file 5: Correlation of Heart Rate with Test Performance**

Project Questionnaire

*The questions below are for research purposes only and will be held strictly confidential.*

1. How anxious are you right now? (1= no stress, 5= very stressed)

*Circle or place mark where appropriate*

1        2           3             4             5

2. How much caffeine did you drink in the past 12 hours? (a drink being about one 8 oz cup of coffee or one 16 oz soda)

*Mark the best approximation*

___ Less than one caffeinated beverage

___ From 1 to 3 caffeinated beverages

___ More than 3 caffeinated beverages

3. How much caffeine do you drink in an average 24 hour period? (a drink being about one 8 oz cup of coffee or one 16 oz soda)

*Mark the best approximation*

___ Less than one caffeinated beverage

___ From 1 to 3 caffeinated beverages

___ More than 3 caffeinated beverages

4. Did you take any medicine or drugs that could affect your heart rate for this exercise?

*Circle*

Yes / No / Unsure

If so, what?:

**Thank you very much for your participation!**
